# Modified Mesoporous Carbon Material (Pb-N-CMK-3) Obtained by a Hard-Templating Route, Dicyandiamide Impregnation and Electrochemical Lead Particles Deposition as an Electrode Material for the U(VI) Ultratrace Determination

**DOI:** 10.3390/ma14216490

**Published:** 2021-10-29

**Authors:** Katarzyna Tyszczuk-Rotko, Rafał Olchowski, Jędrzej Kozak, Olga Sekerzh-Zenkovich, Ryszard Dobrowolski

**Affiliations:** Faculty of Chemistry, Institute of Chemical Sciences, Maria Curie-Skłodowska University, 20-031 Lublin, Poland; rafal.olchowski@poczta.umcs.lublin.pl (R.O.); jedrekkozak@onet.pl (J.K.); olga.sz11186@gmail.com (O.S.-Z.); ryszard.dobrowolski@mail.umcs.pl (R.D.)

**Keywords:** dicyandiamide-impregnated mesoporous carbon, electrochemically lead film deposited, voltammetric sensor, ultratrace U(V) determination

## Abstract

In this paper, a dicyandiamide-impregnated mesoporous carbon (N-CMK-3), electrochemically modified in situ with lead film (Pb-N-CMK-3), was tested as an electrode material for U(VI) ultratrace determination. The prepared carbon material was characterized by XRD, SEM-EDX, Raman, FT-IR, XPS analysis and nitrogen sorption measurements. The changes of electrochemical properties of glassy carbon electrodes (GCE) after the N-CMK-3 and Pb-N-CMK-3 modification were studied using CV and EIS methods. The modification of the GCE surface by the N-CMK-3 material and Pb film increases the electroactive area of the electrode and decreases the charge transfer residence and is likely responsible for the electrochemical improvement of the U(VI) analytical signal. Using square-wave adsorptive stripping voltammetry (SWAdSV), two linear calibration ranges extending from 0.05 to 1.0 nM and from 1.0 to 10.0 nM were observed, coupled with the detection and quantification limits of 0.014 and 0.047 nM, respectively. The Pb-N-CMK-3/GCE was successfully applied for U(VI) determination in reference materials (estuarine water SLEW-3 and trace elements in natural water SRM 1640a).

## 1. Introduction

The existence of uranium in water ecosystems represents a significant concern for public health and environmental pollution [1]. The primary health outcomes of concern documented with respect to uranium are renal, developmental, reproductive, diminished bone growth, and DNA damage [2]. Therefore, accurate and sensitive techniques for environmental uranium monitoring purposes are highly needed [3]. Although various methods can be applied for uranium determination (for example, fluorimetry [4], inductively coupled plasma mass spectrometry [5], inductively coupled plasma optical emission spectrometry [6], neutron activation analysis [7] and radiometric techniques [8]), extensive sample preconcentration and/or separation procedures are required for many of them [9]. Moreover, they can only be applied in laboratory analysis. Compared to these methods, electrochemical techniques, especially stripping voltammetry (SV), are characterized by simplicity, sensitivity, portable instrumentation and low cost.

Till now, a number of SV procedures and/or electrode materials for U(VI) determination have been developed [10,11,12,13,14]. One of the most popular sensors involved for U(VI) determination is the hanging mercury drop electrode (HMDE), which has many advantages, but it is rarely applied because of the volatility and toxicity of mercury [10]. In 2007, the lead film electrode was proposed for the first time as an alternative to mercury electrodes for U(VI) quantification because of its more environmentally friendly character, possibility of SV measurements in the deoxygenated solutions and wide potential window [11]. Thus far, several SV procedures with lead-film-based electrodes have been proposed for U(VI) determination [12,13,14]. Among them, the lowest detection of 0.011 nM (for an accumulation time of 480 s for the big electrode and 240 s for the small electrode) was obtained using a double accumulation step on two lead film working electrodes [14]. It is also the lowest detection limit obtained using mercury-free electrodes and stripping voltammetry.

Ordered mesoporous carbons of CMK-3-type possess high specific surface area, large pore volume and ordered mesoporous structure, which allows to obtain a novel hybrid material by the chemical modification of its surface. CMK-3-type carbons are also known from their high chemical, thermal and mechanical stability. Moreover, these materials are characterized by the excellent electrical conductivity, which can be additionally enhanced by the insertion of various heteroatoms (e.g., oxygen, nitrogen) onto the carbonaceous surface. Nitrogen and oxygen doped CMK-3-type carbons can be successfully applied for creating electrodes with improved electrochemical properties [15,16].

Herein, we prepared a dicyandiamide-impregnated mesoporous carbon (N-CMK-3) that was utilized to modify a glassy carbon electrode (GCE) surface additionally covered during analysis by lead particles. The new sensor (Pb-N-CMK-3/GCE) was applied for voltammetric assay of U(VI) in environmental water ecosystems. The combination of Pb-N-CMK-3/GCE with AdSV resulted in a simple, sensitive and selective analytical procedure that could be used for the environmental monitoring of U(VI).

## 2. Materials and Methods

### 2.1. Apparatus

*Synthesis of carbonaceous material:* magnetic stirrer equipped with heating plate (Wigo, Pruszków, Poland), laboratory oven (Pol-Eko Apparatus, Wodzisław Śląski, Poland), homemade quartz tubular furnace and Elpin Plus water bath orbital shaker (Elpin Plus, Lubawa, Poland).

*Physicochemical characteristic of carbonaceous material:* The nitrogen adsorption/desorption experiments were conducted at −196 °C with ASAP 2420 analyzer (Micromeritics Inc., Norcross, GA, USA). The sample was degassed at 120 °C in vacuum for 12 h before measurements. The BET surface area (S_BET_), total pore volume (V_T_) and BJH pore size distribution (PSD) were estimated using the desorption branch of the nitrogen adsorption/desorption isotherm. The X-ray diffraction pattern was recorded by an Empyrean (PANalytical, Malvern, UK) diffractometer (CuKαradiation) working with 0.02° size step and 10 s time step. The Raman spectrum was recorded on an inVia Reflex (Renishaw, Wotton-under-Edge, UK) dispersive Raman microscope with an ion-argon laser (514 nm, 20 mW). The morphology studies and elemental analysis measurements were performed by the scanning electron microscope (SEM) Carl Zeiss Ultra Plus (Carl Zeiss, Jena, Germany) equipped with an energy dispersive X-ray (EDX) detector BrukerAXS (Bruker, Karlsruhe, Germany). The SEM microscope was also equipped with secondary electron (SE) and backscattered electron (BSE) detectors. All experiments were carried out under required conditions (20-kV acceleration voltage and 5-nA probe current). The CHN elemental analysis was performed with EA 3000 Elemental Analyzer (Euro Vector, Pavia, Italy). The Fourier-transform infrared (FT-IR) spectra were recorded by FT-IR Nicolet 8700 A spectrometer (Thermo Scientific, Waltham, MA, USA) in the wavenumber range of 400–4000 cm^−1^ using KBr pellets. X-ray photoelectron spectra (XPS) were recorded by using a Multi-Chamber Analytical System (Prevac, Rogów, Poland) equipped with monochromatic K_α_-Al radiation (1486.6 eV) (Gammadata Scienta, Uppsala, Sweden) and an X-ray power of 450 W. The carbon C1s peak at 285 eV was a reference for all binding energies. The zeta potential measurements were carried out by Zetasizer Nano ZS (Malvern Instruments, Malvern, UK). Carbonaceous slurries were prepared by adding 2.0 mg of the ground carbon sample in 2 mL of 10^−3^ M KCl. The pH measurements of 5 mL KCl aqueous solution (10^−3^ mol L^−1^) with added carbon sample (20 mg) were performed by using the pH meter CP-401 (Elmetron, Zabrze, Poland) equipped with a glass electrode after suitable calibration.

*Voltammetric studies:* The µAutolab electrochemical analyzers (Eco Chemie, Utrecht, The Netherlands) controlled by GPES 4.9 or FRA 4.9 software were used in voltammetric and electrochemical impedance spectroscopy (EIS) measurements, respectively. The experiments were performed in a classical electrochemical cell containing 10 mL of solution with a three-electrode system (a modified glassy carbon electrode (Pb-N-CMK-3/GCE) with carbon material obtained by a hard-templating route and dicyandiamide impregnation (N-CMK-3), and electrochemical deposited lead particles (Pb)) with the diameter of 1 mm as a working electrode, a platinum wire as a counter electrode and KCl-saturated Ag/AgCl electrode as a reference electrode). The glassy carbon electrode (GCE) was polished daily on silicon carbide paper (SiC-paper, #4000, Buehler, Skovlunde, Denmark) and alumina particles suspension (1.0, 0.3 and 0.05 µm) on a Buehler polishing pad with subsequent washing and sonication for 30 s. The water reference materials were mineralized for 3 h using a UV-digester made by Mineral, Warszawa, Poland.

### 2.2. Reagents and Solutions

*During synthesis of carbonaceous materials, the following chemicals were used:* non-ionic block copolymer Pluronic P123 purchased from Sigma-Aldrich, St. Louis, MO, USA; tetraethoxysilane (TEOS, 98%) and dicyandiamide (99%) purchased from Alfa Aesar, Haverhill, MA, USA; hydrochloric acid (36%), ethanol (99.8%) and sodium hydroxide (99%) purchased from POCH, Polish Chemical Reagents, Gliwice, Poland; sulfuric acid (96%) purchased from Merck KGaA, Darmstadt, Germany; sucrose (food sugar) purchased from Pfeifer and Langen GmbH & Co. KG, Cologne, Germany. All the above-mentioned chemicals were used without additional purification. The ultra-purified (>18 MΩ cm) Milli-Q water (Merck Millipore, Darmstadt, Germany) was used throughout all work.

*During electrochemical measurements, the following chemicals were used:* the buffer solution (0.385 M CH_3_COONH_4_, 0.615 M CH_3_COOH and 0.615 M NH_4_Cl) of pH = 4.2 ± 0.1 obtained by mixing the appropriate volumes of 1 M CH_3_COONH_4_ and 1 M HCl (Sigma-Aldrich, St. Louis, MO, USA), 0.02 M cupferron (N-nitrosophenylhydroxylamine ammonium salt, Merck, Darmstadt, Germany) solution prepared every day by dissolving the reagent in water, the stock solution of U(VI) prepared by dissolving the standard solution (Merck, Darmstadt, Germany) in 0.01 M HNO_3_ and 0.01 M Pb(NO_3_)_2_ solution obtained from Merck (Darmstadt, Germany) reagent. The interference effect was checked using standard solutions of Zn(II), Co(II), Cu(II), Cd(II), Mn(II), Fe(III), Al(III), V(V), Mo(VI), Ni(II) and Triton X-100 (Merck, Darmstadt, Germany). Certified reference material, estuarine water SLEW-3, was obtained from the National Research Council, Canada. Standard reference material, trace elements in natural water SRM 1640a was purchased from the National Institute of Standards and Technology, United States of America.

### 2.3. Synthesis of N-CMK-3

Firstly, the CMK-3-type carbon was synthesized by a hard-templating route according to the procedure already described [15]. Next, the 1 g of CMK-3 was added to the 120 mL of 3.4 wt. % aqueous dicyandiamide solution and the mixture was stirred at 25 °C overnight. The dicyandiamide-impregnated CMK-3 (N-CMK-3) was filtered, dried at 120 °C for 24 h and pyrolyzed in a quartz tubular furnace at 700 °C for 1 h under nitrogen flow (1 L min^−1^).

### 2.4. Preparation of Pb-N-CMK-3/GCE and U(VI) Analysis

Initially, 0.5 mg of N-CMK-3 was dispersed in 100 µL of dimethylformamide (DMF) by shaking at 1000 rpm for 60 s. Then, the N-CMK-3/GCE was prepared by casting 1 µL of the suspension on the polished GCE surface. Afterward, the solvent was allowed to evaporate at room temperature for 180 s.

The square-wave adsorptive stripping voltammetric (SWAdSV) analysis of U(VI) under optimized conditions were carried out in 10 mL of buffer solution (0.385 M CH_3_COONH_4_, 0.615 M CH_3_COOH and 0.615 M NH_4_Cl) of pH = 4.2 ± 0.1 containing 25.0 µM Pb(NO_3_)_2_ and 0.08 mM cupferron. The electrochemical cleaning of the electrode surface was performed at the potential of −1.0 V for 10 s, and then 0.2 V for 10 s. Next, at the potential of −0.8 V (E_dep and acc_) for 180 s (t_dep and acc_), simultaneously, the lead particles were electrochemically deposited at the N-CMK-3/GCE surface and U(VI)-cupferron complexes were accumulated at the Pb-N-CMK-3/GCE surface. The SWAdSV curves were recorded in the potential range from −0.65 to −1.1 V with a frequency (f) of 100 Hz, an amplitude (E_SW_) of 100 mV, a step potential (ΔE) of 10 mV.

## 3. Results and Discussion

### 3.1. Characterization of the CMK-3 and N-CMK-3

**Figure 1:**
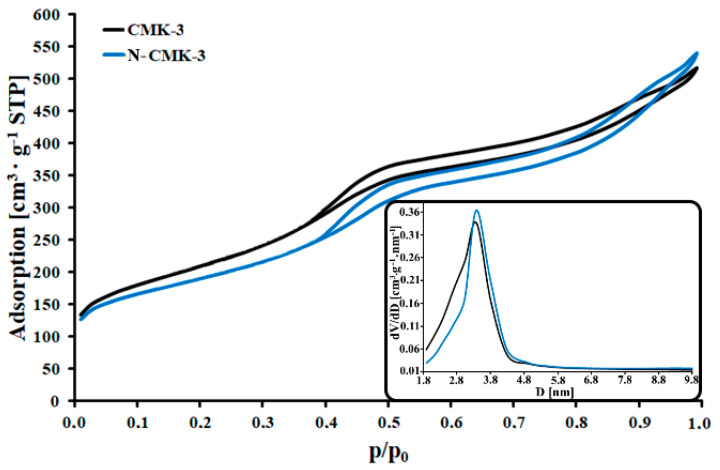
Nitrogen isotherms and pore size distributions (inset) of CMK-3 and N-CMK-3.

In Figure 1, the nitrogen adsorption/desorption isotherms and pore size distributions of both CMK-3 and N-CMK-3 carbon materials are depicted. Presented isotherms are IVa type according to the IUPAC. In each case there is the hysteresis loop of H1 type observed, which appears at 0.38 p/p_0_ (CMK-3) or at 0.4 p/p_0_ (N-CMK-3) [17]. Both the shape of nitrogen isotherms and the presence of a hysteresis loop represent the micro-mesoporous structure of synthesized materials with ordered narrow mesopores. Additionally, the presence of mesopores in the porous structure of CMK-3 and N-CMK-3 carbons is confirmed by pore size distribution results. It can be seen that pores with a diameter of 3.4 nm are the largest population of all present pores in both carbon materials. There are also other pores present with both smaller and larger diameters than 3.4 nm, whose contribution to the overall porous structure of the studied samples is significant. The dicyandiamide thermochemical modification of CMK-3 carbon caused the partial vanishing of pores with a diameter lower than 3.4 nm and simultaneous widening of mesopores with pore diameter higher than 3.4 nm. It can be related to the partial filling of some smaller mesopores with large polymerization products of dicyandiamide, such as melem [18,19] and fragmentary thermal decomposition of oxygen groups from the carbon surface. Moreover, in Table 1 there are presented structural data (S_BET_, V_T_ and d_BJH_) for CMK-3 and N-CMK-3 materials. Studied samples possess a high specific surface area, which suggests the presence of micropores in its porous structures. On the other hand, high V_T_ values and d_BJH_ > 2 nm constitute another confirmation of the presence of mesopores in carbonaceous frameworks of both studied samples. Furthermore, the specific surface area of N-CMK-3 (669 m^2^/g) is lower than for CMK-3 (744 m^2^/g) and the total pore volume of N-CMK-3 (0.83 cm^3^/g) is only slightly higher than in the case of CMK-3 (0.80 cm^3^/g). This is confirmation that the above-mentioned processes can occur during dicyandiamide modification of CMK-3 carbon material.

**Figure 2:**
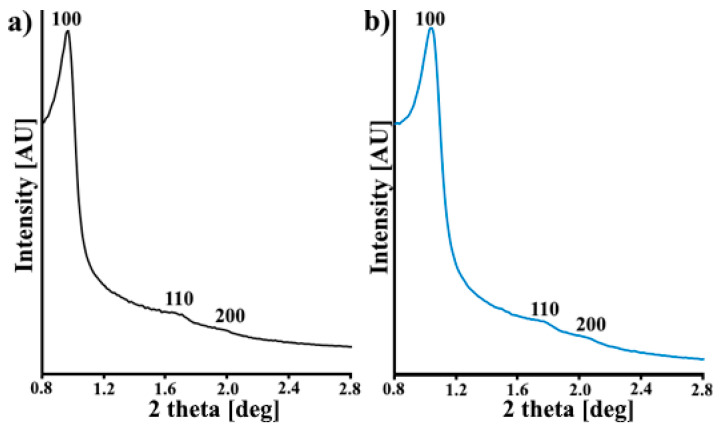
XRD diffractograms of (**a**) CMK-3 and (**b**) N-CMK-3.

In Figure 2, the XRD patterns for the studied samples are presented. Three diffraction peaks indexed to (100), (110) and (200) in the 2 theta angle range from 0.8° to 2.8° are observed for both CMK-3 and N-CMK-3. In each case, the XRD pattern also represents the well-ordered hexagonal mesopores (p6mm symmetry) [20]. The highly ordered mesoporous structure of pristine carbon is retained after dicyandiamide modification.

**Figure 3:**
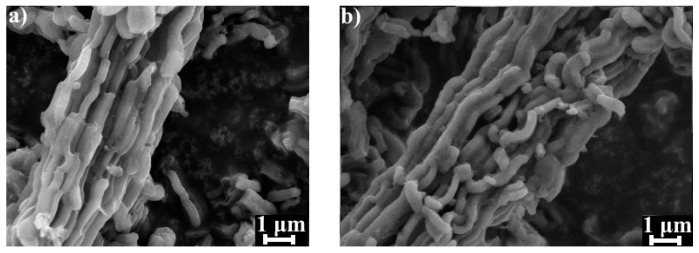
SEM microphotographs (magnification 10,000×) of (**a**) CMK-3 and (**b**) N-CMK-3.

SEM microphotographs of studied carbon materials are depicted in Figure 3. It can be seen that both carbon materials consist of nanoparticles with undisturbed rod-shaped morphology [21]. It confirms that no morphological changes occurred during dicyandiamide modification of pristine CMK-3-type carbon.

**Figure 4:**
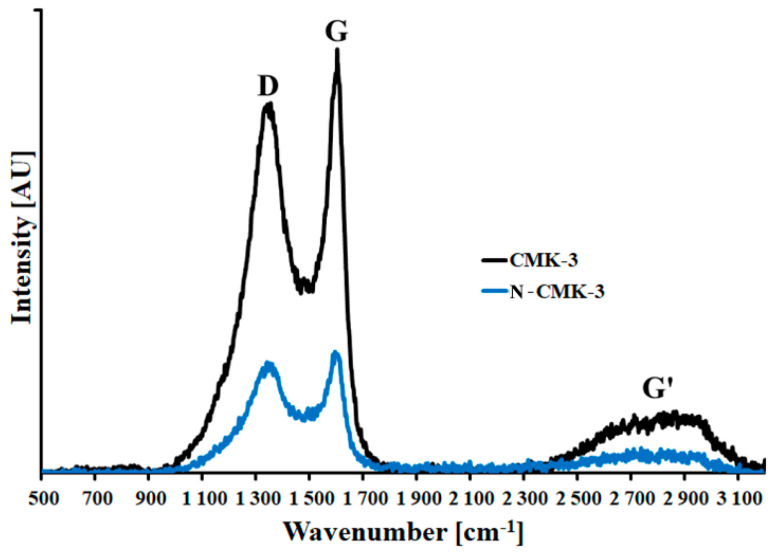
Raman spectra of studied carbon materials.

Raman spectra of CMK-3 and N-CMK-3 carbon samples are presented in Figure 4. In the range of 900–1900 cm^−1^ there are two well-resolved peaks located at 1310 cm^−1^ (D band) and 1580 cm^−1^ (G band). Outside this range there is also a G’ band located at 2800 cm^−1^. The G band originates from vibrations of C=C bonds (sp^2^ hybridization) in unaffected graphene layers, and the D band is related to structural defects of graphene domains. Moreover, the G’ band is an overtone of the G band. The intensity ratio of the D and G bands (I_D_/I_G_) depends on the order degree of the graphene domains in the carbon structure. I_D_/I_G_ << 1 characterizes graphite and unaffected graphene layers. In the case of amorphous carbon, which consists of small disordered graphene layers, the I_D_/I_G_ ≈ 1 [22]. The studied carbonaceous samples are built from an amorphous carbonaceous phase because I_D_/I_G_ values are close to 1 (Table 1). Moreover, the I_D_/I_G_ value for N-CMK-3 carbon is higher than for the pristine one. It suggests the additional deterioration of graphene domains after dicyandiamide modification. This structural disorder of graphene domains can be described by the formation of new surface groups at the edges of graphene layers and the breakdown of already existing surface oxygen groups.

In Table 1, there are also data of zeta potential and pH_surface_ values of studied carbon materials. Negative values of zeta potential (−20.7 mV) and pH_surface_ below 7 (4.6) for CMK-3 carbon suggest the presence of oxygen acidic groups, such as carboxyl, carbonyl and hydroxyl groups. In turn, N-CMK-3 carbon is characterized by the positive value of zeta potential (7.3 mV) and value of pH_surface_ (6.6) close to 7, which can be related to the presence of surface acidic and basic groups with oxygen and nitrogen atoms such as carbonyl, hydroxyl or amide originated from carbon precursor and dicyandiamide modifier [20]. During the polymerization of dicyandiamide, reactive compounds are formed [18,19], which can react with surface oxygen groups. As a result, the formation of nitrogen and oxygen groups and partial decomposition of already existing oxygen groups can occur. The nitrogen basic groups introduced into the structure of pristine CMK-3-type carbon and oxygen basic groups, which are able to adsorb protons from an aqueous solution, can be responsible for the positive charge of the zeta potential and the pH_surface_ value close to neutral. It is worth mentioning that, despite the presence of nitrogen groups, the pH_surface_ of N-CMK-3 does not exceed 7 probably because of remaining acidic oxygen groups on the carbon surface.

Further investigations of surface groups present in CMK-3 and N-CMK-3 carbon materials can be carried out by analyzing results from elemental analysis (CHN, XPS, EDX) and FT-IR studies. In Table 2, the results obtained from elemental analysis of studied carbons are presented. CHN measurements confirmed that the main component of studied samples is carbon (89.0–92.6 wt. %). Furthermore, the dicyandiamide modification of the pristine carbon is responsible for the nitrogen introduction into the carbonaceous structure (nitrogen content change from 0.3 wt. % to 5.9 wt. %). The results from complementary X-ray methods (XPS and EDX) also indicate that carbon is the main component of studied samples. According to EDX results, the CMK-3 carbon also contained oxygen and sulfur. The N-CMK-3 carbon contained three types of heteroatoms (oxygen, nitrogen and sulfur). The sulfur and nitrogen were not detected by XPS in both studied carbon materials. Residual content of sulfur species in the studied samples can originate from the sulfuric acid used during the synthesis procedure of pristine carbon. Interestingly, according to the obtained data, the nitrogen is present in the deeper layers of N-CMK-3 material, which is totally out of the measurement range for the XPS method (maximum a few nm depth) [15]. It can be related to the limited diffusion of dicyandiamide polymerization products to the surface layers of carbonaceous material during the thermochemical modification process. It can also be seen that the dicyandiamide modification of the pristine carbon causes a partial decrease in the oxygen content (from 7.2 wt. % to 4.9 wt. % (XPS data); from 5.5 wt. % to 3.4 wt. % (EDX data)) and increase in nitrogen content (from 0.0 wt. % to 1.5 wt. % (EDX data)). This confirms the above-mentioned considerations about the presence of both acidic oxygen surface groups and basic nitrogen surface groups.

**Figure 5:**
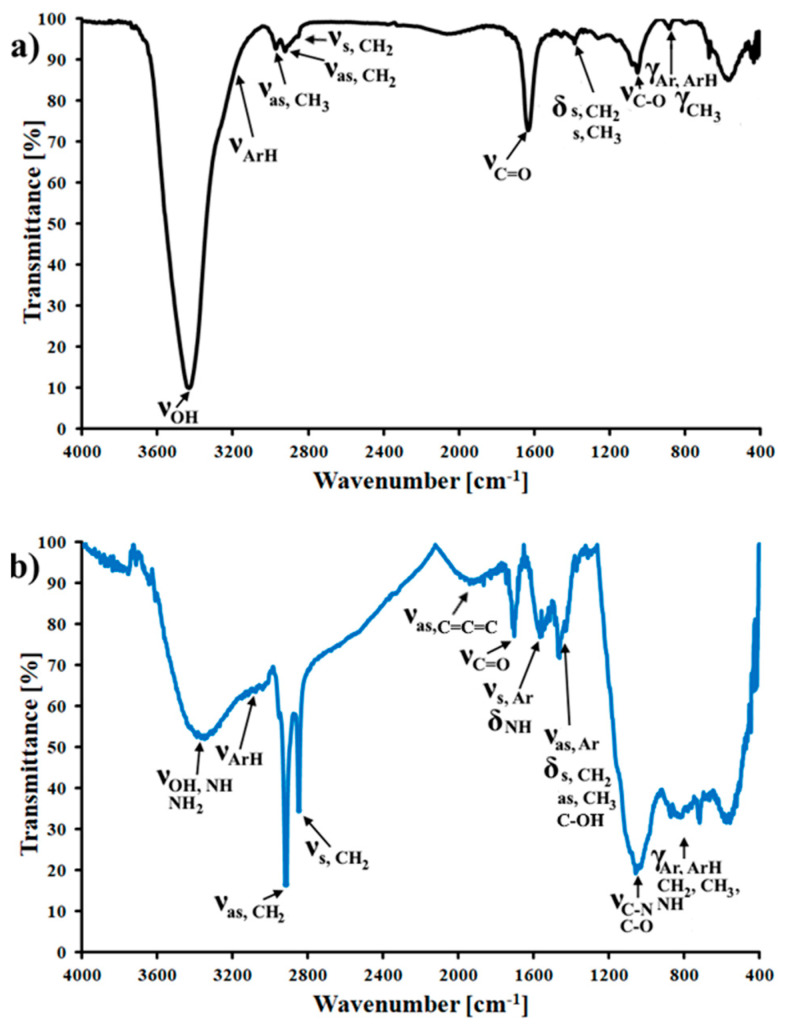
FT-IR spectra of (**a**) CMK-3 and (**b**) N-CMK-3.

More detailed information about the character of surface groups present in synthesized carbons was obtained by FT-IR studies. In Figure 5 FT-IR spectra of CMK-3 and N-CMK-3 carbons are presented. FT-IR spectrum for CMK-3 carbon (Figure 5a) consists of several bands located at 3429 cm^−1^ (ν_OH_), 3000–3100 cm^−1^ (ν_ArH_), 2970 cm^−1^ (ν_as,CH3_), 2920 cm^−1^ (ν_as,CH2_), 2852 cm^−1^ (ν_s,CH2_), 1629 cm^−1^ (ν_C=O_), 1383 cm^−1^ (δ_s,CH2,CH3_), 1047 cm^−1^ (ν_C-O_) and 882 cm^−1^ (γ_Ar,ArH,CH3_). In turn, on the FT-IR spectrum for N-CMK-3 carbon material (Figure 5b) following bands were detected: ν_OH,NH,NH2_ (3350 cm^−1^), ν_ArH_ (3000–3100 cm^−1^), ν_as,CH2_ (2917 cm^−1^), ν_s,CH2_ (2849 cm^−1^), ν_as,C=C=C_ (1916 cm^−1^), ν_C=O_ (1705 cm^−1^), ν_s,Ar_ and δ_NH_ (1568 cm^−1^), ν_as,Ar_, δ_s,CH2_, δ_as,CH3_, δ_C-OH_ (1464 cm^−1^), ν_C-O,C-N_ (1056 cm^−1^) and γ_Ar,ArH,CH3,CH2_ (720–873 cm^−1^). It can be seen that the intensity of the band related to ν_OH_ decreased after dicyandiamide modification of pristine carbon. Simultaneously the intensity of other bands corresponding to oxygen groups but also to aromatic carbon rings, alkyl carbon chains and C=C=C groups increased. Additionally, the vibrations related to the amine and amide groups can be also present on the FT-IR spectrum of N-CMK-3 carbon (vibrations of N-H and C-N groups), although these bands can be overlapped with other bands presented on the FT-IR spectrum of N-CMK-3 carbon. Probably during dicyandiamide modification of CMK-3 carbon some OH groups are removed and nitrogen atoms are introduced to graphene domains of carbonaceous material, which causes the partial deterioration of graphene plains. It can be assumed that the modification of CMK-3 carbon by dicyandiamide molecules caused substantial changes of carbonaceous surface.

### 3.2. Characterization of the N-CMK-3/GCE and Pb-N-CMK/GCE

**Figure 6:**
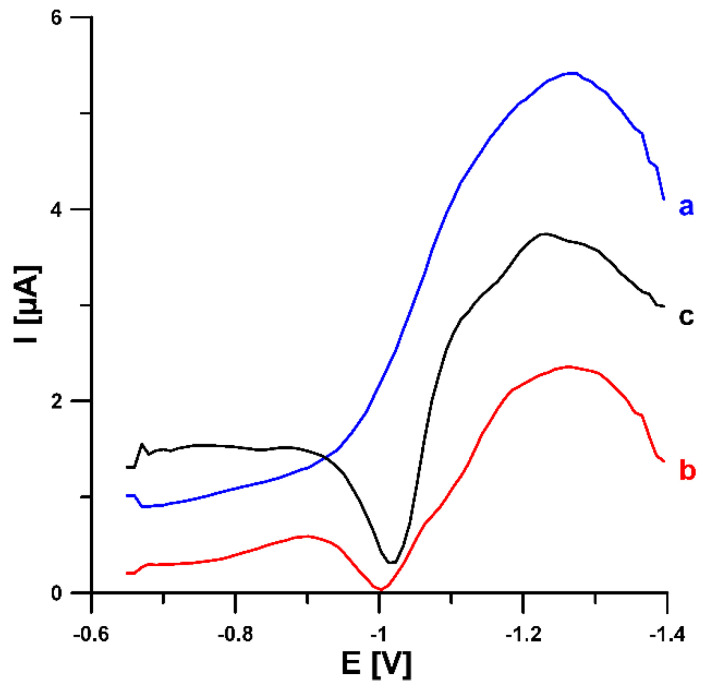
SWAdSVs of 10.0 nM U(VI) obtained at the N-CMK-3/GCE (a), PbF/GCE (b) and Pb-N-CMK-3/GCE (c) in 0.2 M acetate buffer of pH 4.0 containing 0.025 µM Pb(NO_3_)_2_ (b and c) and 0.06 mM cupferron. The SWAdSV parameters: E_dep_ of −1.1 V, t_dep_ of 60 s, E_acc_ of −0.65 V, t_acc_ of 180 s, f of 200 Hz and E_SW_ of 25 mV.

**Figure 7:**
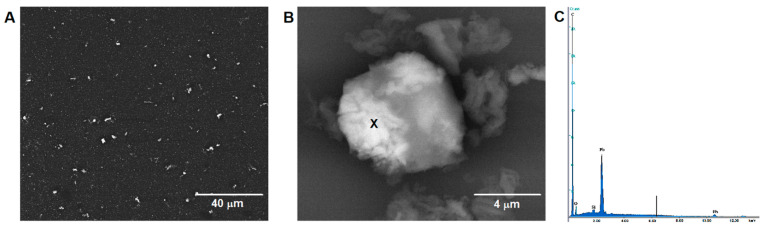
SEM images of Pb-N-CMK-3/GCE (**A**,**B**). (**C**) The EDS spectrum of the highlighted fragment of Pb-N-CMK-3/GCE.

The obtained N-CMK-3 material doped with electron-rich oxygen and nitrogen atoms attached to the surface of hexagonally ordered nanorods and plenty of structural defects should improve the electron transfer of the N-CMK-3-based electrode. To check these assumptions, the electrochemical activity of N-CMK-3/GCE towards the reduction of U(VI)-cupferron complexes was analyzed using the SWAdSV technique. Figure 5 shows the SWAdSV responses of 10.0 nM U(VI) at the GCE modified with lead film (PbF/GCE), and N-CMK-3/GCE and N-CMK-3/GCE modified in situ with lead particles (Pb-N-CMK-3/GCE). All electrodes show a clear reduction wave. However, the analytical signal at the N-CMK-3/GCE is stretched. These analytical signals at the potential around −1.0 V (−0.96 V for N-CMK-3/GCE, −1.03 V for PbF/GCE, −1.0 V for Pb-N-CMK-3/GCE) are connected with the U(VI) reduction process, which is well described in the literature [23]. As shown in Figure 6, the U(VI)-cupferron complexes are effectively reduced at the PbF/GCE and N-CMK-3/GCE, giving rise to a peak current of 0.75 and 0.88 µA. When the Pb-N-CMK-3 was used as a modifier of a GCE, the reduction peak current of U(VI) was increased to 2.10 µA. These results clearly show that a dicyandiamide-impregnated mesoporous carbon (N-CMK-3) electrochemically modified in situ with lead film (Pb-N-CMK-3) acts as the best electron conducting mediator between the electrode surface and the supporting electrolyte and facilitates the electrochemical reduction of U(VI). It is most likely that the developed porosity as well as surface chemistry of N-CMK-3 facilitates Pb modification of the electrode surface. This further translates into an increase in the number of active sites on which the U(VI)-cupferron complexes can be adsorbed. Figure 7 shows SEM images of the Pb-N-CMK-3/GCE surface. As can be seen in Figure 7A, the surface of the N-CMK-3/GCE is covered with bright points, with the equivalent diameter being in the range of 1.3–7.3 μm. Higher magnification revealed that these bright points are surrounded by N-CMK-3 material (Figure 7B). The energy dispersive spectrometry spectrum corresponding to the SEM image is given in Figure 7C. As can be seen, the presence of lead is manifested by signals on the EDS spectrum.

**Figure 8:**
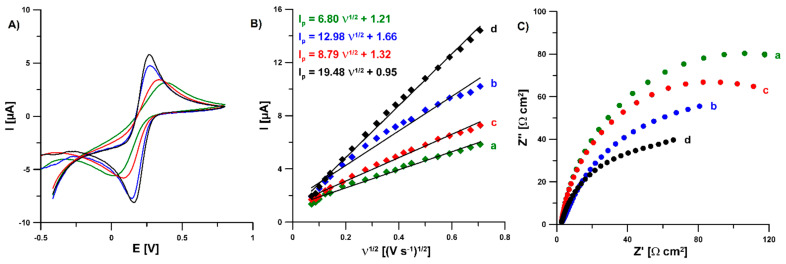
(**A**) CVs recorded in 0.1 M KCl containing 5.0 mM K_3_[Fe(CN)_6_] at the bare GCE (green curve), PbF/GCE (red curve), N-CMK-3/GCE (blue curve) and Pb-N-CMK-3/GCE (black curve) at the scan rate of 100 mV s^−1^. (**B**) The dependence between I_p_ and υ^1/2^ for the bare GCE (a), PbF/GCE (c), N-CMK-3/GCE (b) and Pb-N-CMK-3/GCE (d). (**C**) Nyquist plots of the bare GCE (a), PbF/GCE (c), N-CMK-3/GCE (b) and Pb-N-CMK-3/GCE (d) recorded at a potential of 0.2 V, in the frequency range from 10 kHz to 0.1 Hz.

To investigate the electrochemical properties of Pb-N-CMK-3/GCE, cyclic voltametric (CV) and electrochemical impedance spectroscopic (EIS) studies in a solution of 0.1 M KCl containing 5.0 mM K_3_[Fe(CN)_6_] were performed. To compare the results obtained with the Pb-N-CMK-3/GCE, CV and EIS measurements were also performed at the bare GCE, the PbF/GCE and the N-CMK-3/GCE. Figure 8A shows the CVs recorded at a scan rate of 100 mV s^−1^ on all tested electrodes, and well-defined redox peaks currents were noted in each electrode. The relative peak separations (χ^0^, χ^0^ = (E_pa_ − E_pc_)/0.058) calculated for the GCE, PbF/GCE, N-CMK-3/GCE and Pb-N-CMK-3/GCE are equal to 5.54, 3.84, 2.06 and 1.78, respectively. The results indicate that the Pb-N-CMK-3/GCE shows the fastest electron kinetics. Figure 8B shows the dependence between anodic peak current (I_p_) and the square root of the scan rate (υ^1/2^) for all studied electrodes. For the GCE, PbF/GCE, N-CMK-3/GCE and Pb-N-CMK-3/GCE, the active surface areas (A_s_) are equal to 0.0019, 0.0024, 0.0037 and 0.0054 cm^2^, respectively, while the geometric area of GCE is equal to 0.0314 cm^2^ [24]. As can be seen, the A_s_ is the largest for the Pb-N-CMK-3/GCE. Furthermore, the A_s_ for the GCE, PbF/GCE, N-CMK-3/GCE and Pb-N-CMK-3/GCE is smaller than the geometric area, which could be explained by the presence of the insulating resin in the interior of the GCE. Figure 8C shows Nyquist plots recorded in the frequency range from 1 MHz to 0.1 Hz at a potential of 0.2 V. The obtained results showed that the charge transfer resistance (R_ct_) of Pb-N-CMK-3/GCE (104 Ω) is significantly smaller than that for the GCE (204 Ω), and also for PbF/GCE (172 Ω) and N-CMK-3/GCE (161 Ω). It is connected with the presence of conductive modifiers on the GCE surface.

### 3.3. Optimization of Experimental Conditions

To establish the optimal conditions for voltametric determination of U(VI) at the Pb-N-CMK-3/GCE, thoroughgoing studies were carried out. Firstly, in order to improve the electrochemical response of the Pb-N-CMK-3/GCE towards U(VI) determination, the supporting electrolyte composition (CH_3_COONH_4_, CH_3_COOH and NH_4_Cl) was proposed [13]. Then, the effect of pH buffer over the range from 3.5 to 6.0 was investigated. The maximum U(VI) peak current (5.0 nM U(VI)) was observed at a pH of 4.2 ± 0.1 and this value was considered suitable for further studies. Furthermore, the effect of Pb(NO_3_)_2_ as well as cupferron concentration on the 5.0 nM U(VI) signal was studied in the range of 5.0 –75.0 µM and 0.01–0.1 mM, respectively. The highest analytical signal of U(VI) was obtained for 25.0 µM Pb(NO_3_)_2_ solution and 0.08 mM cupferron.

To improve the sensitivity and detection limit of the analytical procedure, the deposition potential (E_dep_) and time (t_dep_) of lead particles of the N-CMK-3/GCE surface as well as the accumulation potential (E_acc_) and time (t_acc_) of U(VI)-cupferron complexes on the Pb-N-CMK-3/GCE surface were investigated. The effect of E_dep_ on the reduction peak current of U(VI) (5.0 nM U(VI)) was studied in the range from −0.6 to −1.1 V (t_dep_ of 60 s, E_acc_ of −0.65 V, t_acc_ of 180 s). It was found that the current response of U(VI) increased with the E_dep_ from −0.6 to −0.8 V. Therefore, the E_dep_ of −0.8 V was selected for further experiments. Next, the effect of E_acc_ on the reduction peak current of U(VI) (5.0 nM U(VI)) was studied in the range from −0.4 to −1.1 V (E_dep_ of −0.8 V, t_dep_ of 60 s, t_acc_ of 180 s). The U(VI) signal increased strongly, reaching a maximum at a potential of −0.8 V. Consequently, the lead particles deposition and the U(VI)-cupferron complexes accumulation were performed simultaneously at the potential −0.8 V (E_dep and acc_). Moreover, the effect of time of simultaneous deposition of lead and U(VI)-cupferron complexes accumulation (t_dep and acc_) in the range of 15–300 s was investigated. It was found that the U(VI) peak current was highly sensitive to the t_dep and acc_. The current response increased with the t_dep and acc_ up to 300 s. However, in order to reduce analysis time, the t_dep and acc_ of 180 s was selected for further experiments.

Further, the optimization of SWV parameters (the SW frequency—f, the SW amplitude—E_sw_, the step potential—ΔE) was performed. During adjustment of the above-mentioned parameters, each of them was changed, while the others were kept fixed using 5.0 nM U(VI) concentration. For E_sw_ of 25 mV and ΔE of 10 mV, the SW frequency was varied from 25 to 200 Hz. The highest U(VI) signal was obtained at the f of 200 Hz. However, the f higher than 100 Hz caused a distorted peak shape. Therefore, the f equal to 100 Hz was applied in the subsequent study. Then, the effect of the SW amplitude, ranging from 25 to 125 mV was tested. The maximum peak height accompanied by the best signal was obtained for the E_sw_ of 100 mV, thus this value was chosen for the subsequent study. Moreover, the step potential of the staircase waveform was varied from 1 to 10 mV. For ΔE of 10 mV, the highest U(VI) peak current was attained.

### 3.4. Analytical Characteristic of Pb-N-CMK-3/GCE towards U(VI)

**Figure 9:**
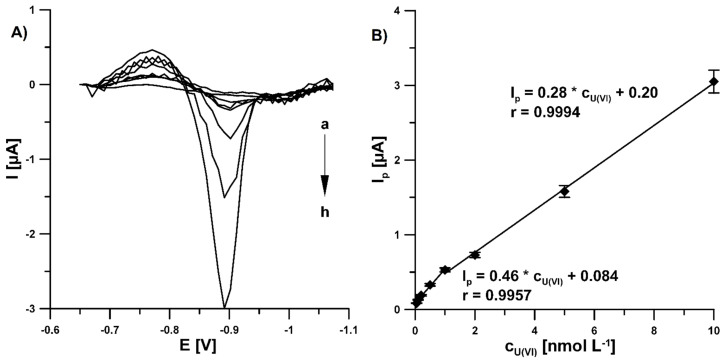
(**A**) SWAdSVs recorded at the Pb-N-CMK-3/GCE in the buffer solution (0.385 M CH_3_COONH_4_, 0.615 M CH_3_COOH and 0.615 M NH_4_Cl) of pH = 4.2 ± 0.1 containing 25.0 µM Pb(NO_3_)_2_, 0.08 mM cupferron and increasing concentrations U(VI): (a) 0.05, (b) 0.1, (c) 0.2, (d) 0.5, (e) 1.0, (f) 2.0, (g) 5.0, (h) 10.0 nM. (**B**) Calibration plot of U(VI). The SWAdSV parameters: E_dep and acc_ of −0.8 V, t_dep and acc_ of 180 s, f of 100 Hz, E_SW_ of 100 mV and ΔE of 10 mV.

The SWAdSV curves recorded at the Pb-N-CMK-3/GCE under optimized conditions, after the step wise addition of increasing concentrations of U(VI) from 0.05 to 10.0 nM, are displayed in Figure 9A. On plotting the peak reduction current as a function of the U(VI) concentration, two linear relationships were observed over the concentration ranges of 0.05–1.0 nM (I_p_ (µA) = 0.46 × C_U(VI)_ (nM) + 0.084, r = 0.9957) and 1.0–10.0 nM (I_p_ (µA) = 0.28 × C_U(VI)_ (nM) + 0.20, r = 0.9994). The corresponding linear plot is shown in Figure 9B. The detection (LOD) and quantification (LOQ) limits were calculated at 0.014 and 0.047 nM, respectively, (LOD = 3SDa/m and LOQ = 10SDa/m, SDa—standard deviation of intercept (n = 3), m—slope of calibration curve) [25]. The reason for two linear regions may be as follows: at higher concentration than 1.0 nM, saturation took place, and this defined the decrease in the current in the interval from 1.0 to 10.0 nM or the analyte transport mechanism altered. The developed voltametric procedure for the determination of U(VI) offers a much lower limit of detection (0.014 nM) than most other methods: laser fluorimetry (LOD of 12.6 nM) [4], flame atomic absorption spectrometry (LOD of 210 µM) [6], electrothermal atomic absorption spectrometry (LOD of 25.2 nM) [6], inductively coupled plasma optical emission spectrometry (LOD of 2.10 nM) [6], ion chromatography inductively coupled plasma mass spectrometry (LOD of 0,13 nM) [8]. In the case of electrochemical sensors, there are only three procedures in the literature in which the LOD was lower than in this paper [26,27,28]. However, achieving ultra detection limits (5.0, 2.0 and 0.15 pM, respectively) requires the construction of complex recognition strategies, such as DNA amplification and using large surface-area substrates. Those procedures are time- and cost-consuming.

**Figure 10:**
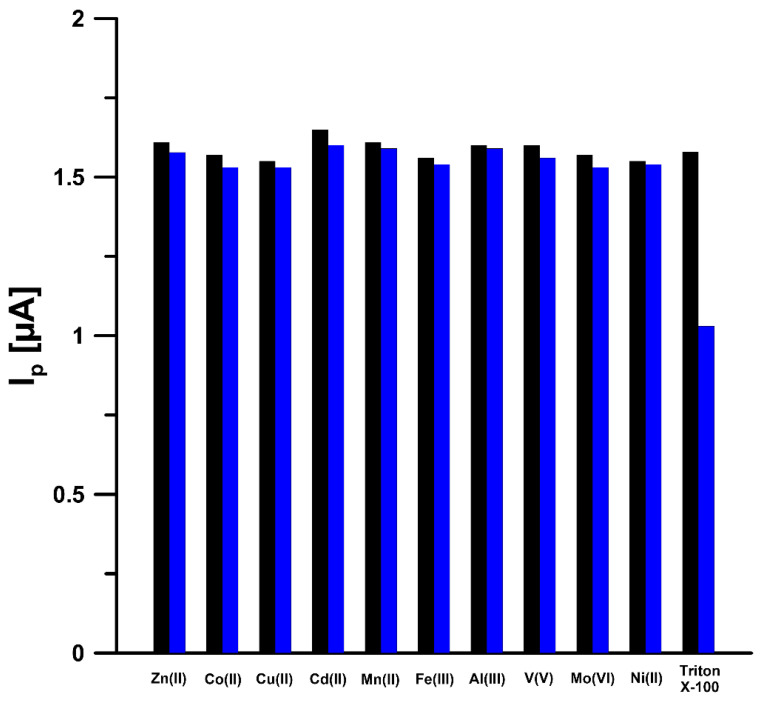
Histogram bars of the U(VI) peak current in the presence of interferents.

Furthermore, the analytical performance of the Pb-N-CMK-3/GCE was studied by considering selectivity, reproducibility and repeatability. To study the selectivity, the SWAdSV with use of Pb-N-CMK-3/GCE was applied for 5.0 nM U(VI) determination in the absence and presence of a number of metal ions (Zn(II), Co(II), Cu(II),Cd(II), Mn(II), Fe(III), Al(III), V(V), Mo(VI), Ni(II)) coexisting in environmental water samples. The concentration of these potential interferents was mentioned at 5.0 nM. All of the interfering metal ions have negligible effects on the U(VI) peak current with the relative standard deviation (RSD) ≤ 3.0%. However, the addition of 1 mg L^−1^ of Triton X-100 generated a decrease in the U(VI) peak to 65% of its original value, so that the mineralization of water samples are recommended before U(VI) analysis (Figure 10).

The U(VI) signal at the Pb-N-CMK-3/GCE exhibits very good repeatability with an RSD of 3.1% (5.0 nM U(VI), n = 10). To investigate the reproducibility of Pb-N-CMK-3/GCE, three different sensors were prepared and employed in the determination of 5.0 nM U(VI). The RSD value was 7.2%, which confirmed the acceptable reproducibility of the Pb-N-CMK-3/GCE.

### 3.5. Application to Reference Materials Analysis

**Figure 11:**
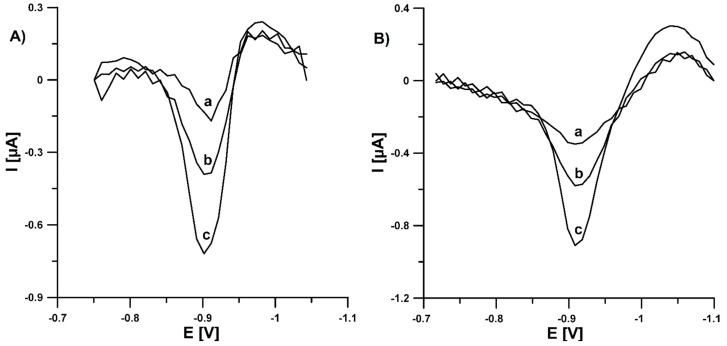
SWAdSVs obtained for the determination of U(VI) in SLEW-3 certified reference material (**A**): (a) 1 mL of sample, (b) as (a) + 1.0, (c) as (a) + 2.0 nM U(VI) and standard reference material 1640a (**B**): 91 µL of sample, (b) as (a) + 1.0, (c) as (a) + 2.0 nM U(VI). The SWAdSV parameters: E_dep and acc_ of −0.8 V, t_dep and acc_ of 180 s, f of 100 Hz, E_SW_ of 100 mV and ΔE of 10 mV.

Subsequently, to check the correctness of the developed SWAdSV procedure with the use of Pb-N-CMK-3/GCE, it was applied to determine U(VI) in the reference materials (estuarine water SLEW-3 and trace elements in natural water SRM 1640a). The determinations were carried out using the standard additions method. Table 3 shows obtained results while Figure 11 shows recorded SWAdSV curves. The relative errors between results obtained by the proposed method and information/certified values are equal to 5.5 and 0.91%. The relative error values as well as the calculated t values (t_exp_) from the Student’s *t*-test prove the high degree of accuracy of the SWAdSV procedure. The t_exp_ values (0.27 and 0.33) do not exceed the critical value (t_crit_, 4.303, level of significance of 0.05, number of degrees of freedom (f) of 2, f = n − 1), and therefore it must be concluded that the measured values are not different from the information/certified value in a statistically significant manner [29].

## 4. Conclusions

The dicyandiamide-impregnated mesoporous carbon (N-CMK-3) doped with electron-rich oxygen and nitrogen atoms attached to the surface of hexagonally ordered nanorods, additionally modified during analysis (in situ method) by lead particles, was successfully applied as electrode material for the ultratrace determination of U(VI). The modification of the glassy carbon electrode (GCE) surface by the Pb-N-CMK-3 material increases the active surface area of the sensor and decreases the charge transfer resistance, which translates into a significant increase in the U(VI) peak current. The proposed electroanalytical method (square-wave adsorptive stripping voltammetry, SWAdSV), performed under optimized conditions, exhibits very low detection and quantification limits (0.014 and 0.047 nM, respectively). Moreover, it offers the sensitive determination of U(VI) with adequate repeatability, reproducibility and selectivity. The elaborated procedure allows for U(VI) determination in environmental water samples without a complicated sample pretreatment step, which was confirmed by satisfactory results obtained during the reference materials analysis (estuarine water SLEW-3 and trace elements in natural water SRM 1640a). Summing up, the SWAdSV with the use of Pb-N-CMK-3/GCE can be a useful tool for the U(VI) determination in water ecosystems.

## Figures and Tables

**Table 1:** Parameters of the porous structure (S_BET_, V_T_ and d_BJH_) obtained from nitrogen isotherms, zeta potential, Raman I_D_/I_G_ and pH_surf._ values for both studied samples.

Parameters of Porous Structure	CMK-3	N-CMK-3
S_BET_ (m^2^/g)	744	669
V_T_ (cm^3^/g)	0.80	0.83
d_BJH_ (nm)	3.4	3.4
Zeta potential/ Raman I_D_/I_G_/pH_surf._	CMK-3	N-CMK-3
ζ (mV)	−20.7	7.3
I_D_/I_G_ (au)	0.87	0.91
pH_surf._ (au)	4.6	6.6

**Table 2:** CHN, XPS and EDX elemental analysis results for CMK-3 and N-CMK-3.

**Measurement method**	**CHN**		**CMK-3**	**N-CMK-3**
		C (wt. %)	92.6	89.0
		H (wt. %)	0.7	0.6
		N (wt. %)	0.3	5.9
	**XPS**	C (wt. %)	92.8	95.1
		O (wt. %)	7.2	4.9
		N (wt. %)	0.0	0.0
		S (wt. %)	0.0	0.0
	**EDX**	C (wt. %)	93.0	94.8
		O (wt. %)	5.5	3.4
		N (wt. %)	0.0	1.5
		S (wt. %)	0.5	0.3

**Table 3:** The results of U(VI) determination in reference materials.

Reference Material	Measured Value ± SD (n = 3) (µM)	Information/Certified Value ± SD (µM)	Relative Error (%) ^c^	t_exp_
SLEW-3	0.00103 ± 0.00038	0.00109 ^a^	5.5%	0.27
SRM 1640a	0.109 ± 0.0053	0.11 ^b^ ± 0.0011	0.91%	0.33

^a^—information value, ^b^—certified value, ^c^—relative error (%) = (information or certified value − measured value)/(information or certified value) × 100%.

## Data Availability

The data presented in this study are available on request from the corresponding author.
